# Differential Transcriptional Regulation of *meis1* by Gfi1b and Its Co-Factors LSD1 and CoREST

**DOI:** 10.1371/journal.pone.0053666

**Published:** 2013-01-07

**Authors:** Asif H. Chowdhury, Johnny R. Ramroop, Ghanshyam Upadhyay, Ananya Sengupta, Anna Andrzejczyk, Shireen Saleque

**Affiliations:** Department of Biology, The City College of New York and The Graduate Center of The City University of New York, New York, New York, United States of America; National University of Singapore, Singapore

## Abstract

Gfi1b (growth factor independence 1b) is a zinc finger transcription factor essential for development of the erythroid and megakaryocytic lineages. To elucidate the mechanism underlying Gfi1b function, potential downstream transcriptional targets were identified by chromatin immunoprecipitation and expression profiling approaches. The combination of these approaches revealed the oncogene *meis1*, which encodes a homeobox protein, as a direct and prominent target of Gfi1b. Examination of the *meis1* promoter sequence revealed multiple Gfi1/1b consensus binding motifs. Distinct regions of the promoter were occupied by Gfi1b and its cofactors LSD1 and CoREST/Rcor1, in erythroid cells but not in the closely related megakaryocyte lineage. Accordingly, Meis1 was significantly upregulated in LSD1 inhibited erythroid cells, but not in megakaryocytes. This lineage specific upregulation in Meis1 expression was accompanied by a parallel increase in di-methyl histone3 lysine4 levels in the Meis1 promoter in LSD1 inhibited, erythroid cells. Meis1 was also substantially upregulated in *gfi1b−/−* fetal liver cells along with its transcriptional partners Pbx1 and several Hox messages. Elevated Meis1 message levels persisted in *gfi1b* mutant fetal liver cells differentiated along the erythroid lineage, relative to wild type. However, cells differentiated along the megakaryocytic lineage, exhibited no difference in Meis1 levels between controls and mutants. Transfection experiments further demonstrated specific repression of *meis1* promoter driven reporters by wild type Gfi1b but neither by a SNAG domain mutant nor by a DNA binding deficient one, thus confirming direct functional regulation of this promoter by the Gfi1b transcriptional complex. Overall, our results demonstrate direct yet differential regulation of *meis1* transcription by Gfi1b in distinct hematopoietic lineages thus revealing it to be a common, albeit lineage specific, target of both Gfi1b and its paralog Gfi1.

## Introduction

Growth factor independence (Gfi)1 and Gfi1b are homologous zinc finger transcriptional repressors that perform critical and essential functions in multiple developmental processes and stages. In the hematopoietic system, Gfi1 is required for maintaining stem cell homeostasis in the bone marrow [1], [2], generating neutrophils [3], [4], and ensuring proper development and maturation of other innate and adaptive lymphoid cells [5], [6], [7]. Gfi1 also controls differentiation of non-hematopoietic tissues including inner ear hair cells, lung, and intestine [8], [9], [10]. Gfi1b is essential for generation of the definitive erythroid and megakaryocytic lineages in the fetal liver [11]. Conditional deletion of *gfi1b* in adult HSCs also perturbs quiescence, resulting in ectopic mobilization and expansion of the HSC compartment [12].

Gfi1 and Gfi1b have also been implicated in oncogenesis. Gfi1 exhibits major oncogenic potential and has been associated with both murine and human cancers [9], [13], [14], [15]. Additionally, over expression of Gfi1 co-operatively accelerates the rate of lymphomagenesis in collaboration with the oncogenes *c-myc* or *pim1* [16]. Similar results have also been reported for Gfi1b [17]. Recent reports also suggest a probable connection between both factors and chronic (CML) and acute (AML) myeloid leukemias [18], [19]. While Gfi1 was shown to be over-expressed in chronic myeloid leukemias in a case study in China [19], a variant of Gfi1 (S36N) (in which the serine residue at position 36 is replaced by an asparagine) was observed to be prevalent in AML patients in Europe [20]. Gfi1b was earlier shown to be over-expressed in erythroid and megakaryocytic malignancies and the proliferative capacity of leukemic cell lines was found to be directly proportional to Gfi1b levels [21]. Full length Gfi1b and a shorter splice variant isoform of it were also shown to be over-expressed in AML and CML patients [18]. The shorter Gfi1b splice variant Gfi1b^p32^ was also recently shown to be expressed in normal cells and required for erythroid differentiation of a multi-potent hematopoietic cell line [22].

Although, ablations of *gfi1* and *gfi1b* function produce both distinct and overlapping phenotypes, these proteins exhibit remarkable physiological and mechanistic interchangeability during hematopoietic development as evidenced by targeted replacement of *gfi1* with *gfi1b* (knock in) in the mouse genome [23], and in the co-factors they associate with to mediate their functions [24], [25], [26], [27]. Knock in of the *gfi1b* cDNA into the *gfi1* locus produced ostensibly normal hematopoietic development but did not rescue inner ear defects ensuing from loss of *gfi1* [23]. Both proteins also associate with the chromatin regulatory factors LSD1 (lysine specific demethylase1), CoREST/Rcor1 (REST co-repressor) and HDACs1–2 (histone deacetylases) via their N terminal, 20 amino acid long, transcriptional repression SNAG domains [24], [27]. Recruitment of these co-factors mediates reversible transcriptional repression of Gfi1 and Gfi1b target genes. Gfi1 and Gfi1b also associate with the histone methyl transferase, G9a via other domains which likely mediates or initiates relatively stable, long term silencing of their targets [25], [26]. The function of their almost identical 20 amino acid long SNAG domains is further underscored by an amino acid substitution (proline to alanine) at position 2 that ablates both transcriptional repression and biological activities of both proteins albeit in distinct cellular contexts [23], [27], [28], [29]. Consistent with their virtually identical, C terminal, DNA binding zinc fingers they also bind the same consensus sequence TAAATCAC(A/T)GCA in the promoters of their gene targets [17], [30]. Thus these proteins are likely to share, and repress, many common targets though the actual pool of responsive targets in any cell type may be determined by cellular context and chromatin accessibility.

Despite the diverse and essential roles of Gfi-1 and Gfi-1b, relatively little is known regarding their mechanism of action, particularly the identity and function of the significant gene targets of Gfi1b and how they mediate the functions of this protein. Therefore, we performed chromatin immunoprecipitation screens (ChIP on chip) to identify common gene targets of Gfi1b and its co-factors LSD1 and CoREST in erythroid cells as previously reported, and identified 653 ChIP targets of all three proteins [27]. To determine the regulation of these targets by Gfi1b/LSD1/CoREST, expression profiling of these genes was performed in control versus LSD1 inhibited erythroid cells since depletion of LSD1 had previously been shown to up-regulate known Gfi1b gene targets (including itself) in erythroid cells [27]. The combination of the ChIP and microarray profiling screens revealed that the oncogene *meis1* (myeloid ecotropic virus integration site1), a three amino acid loop extension (TALE) subclass of homeodomain transcription factors, was most robustly regulated by the Gfi1b complex, following that of its own promoter as previously described [31].

Analysis of the ChIP sequences further demonstrated the presence of 2 distinct *meis1* promoter segments with numerous consensus and quasi-consensus Gfi1/1b sites that were occupied by Gfi1b, LSD1 and COREST specifically in erythroid cells but not in megakaryocytes. Accordingly, inhibition of LSD1 in erythroid cells but not in megakaryocytes lead to elevated Meis1 expression, and was also accompanied by elevated di-methyl H3–K4 levels in the promoter chromatin of the of the former. Meis1 mRNA was also found to be upregulated in *gfi1b−/−* fetal liver cells relative to wild type controls, and this upregulation persisted when these cells were differentiated along the erythroid lineage but disappeared when cultured along the megakaryocytic lineage. The message levels of Meis1 transcriptional partners, and other related proteins, that are often co-ordinately upregulated with it in different leukemias, such as Pbx1 and a number of Hox family members [32], [33] was also found to be elevated in the mutants although to different extents. Finally, different segments of the Meis1 promoter were observed to be repressed by Gfi1b in reporter based transfection assays. This repression required both the intact SNAG and DNA binding domains of Gfi1b.

Our results demonstrating direct regulation of *meis1* transcription by Gfi1b complement a similar relationship between its paralog, Gfi1 and Meis1 expression in myeloid cells [34]. Although this study neither documented direct regulation of the *meis1* promoter by Gfi1, nor association of Gfi1 and its co-factors with the promoter chromatin, nevertheless these results confirm independent, lineage specific, regulation of *meis1* transcription by both Gfi paralogs. This demonstration of *meis1* as a common gene target of both Gfi1 and Gfi1b in turn underscores the functional basis of the observed physiological interchangeability between these proteins. Given that the fly ortholog of the Gfi proteins, Senseless (Sens) opposes Meis/Hox function during fly neurogenesis [35], our results further demonstrate that functional antagonism between the *hox* and *gfi/sens* families has been conserved through evolution from flies to mammals despite duplication of the *sens* orthologs in the latter.

## Materials and Methods

### ChIP and ChIP on Chip

Chromatin immunoprecipitations were performed in MEL and L8057 cells as previously described [27]. Briefly, 5×10^7^ cells were used per ChIP reaction, crosslinked with 1% formaldehyde, sonicated, precleared, and incubated with 5–10 µg of antibody or pre-immune sera. Complexes were washed with low and high salt buffers, and the DNA was extracted and precipitated. Primers used for ChIP were as follows:

Gfi-1Bp: CGCCAGATTTTGACACAAATAA, CTGCACAGACAGACACTTCTCC.

Meis1.1: GATAATTGATTTTCCCCGCAGC, GAAAATGAGCTCACCCAAATCTC.

mMeis1.2: CTGATTTTTTTGGGGGGGAGA, CTGAGCATAAAAGCGCTCTGG.

ChIP on chip was performed as previously described [27]. Hybridization was performed on the Affymetrix GeneChip Mouse promoter 1.0R arrays. MAT [36] was applied to predict the target loci, and targets were predicted at the MAT p value cutoff of 1.0×10^−6^. Mouse genome annotation released in March 2006 (mm8, refFlat) of genomic regions between 5 kb 5′ and 2 kb 3′ of transcription start site (TSS) was searched to predict target sequences. Microarray expression profiling was performed using the Affymetrix Mouse Chip 430_2 array and results compared with the CHIP on chip data.

### Extract Preparation and Western Blotting

Whole cell extracts from different cell lines and Western blotting was performed as previously described [27]. Antibodies used for ChIP and Westerns have also been previously described [27].

### RNAi and qPCR

Mel and L8057control cell lines or those stably expressing LSD1shRNA(TGCTGTTGACAGTGAGCGCGGATGGGATTTGGCAACCTTATAGTGAAGCCACAGATGTATAAGGTTGCCAAATCCCATCCTTGCCTACTGCCTCGGA) from the retroviral vector mIR-PIG (LTR-U6-Mir30-PuroIresGFP) as previously described [27] were used for RNA extraction and qPCR. Total fetal livers were also harvested from e12.5 mouse embryos and used for RNA extraction. RNA was prepared from cell lines and primary cells using the RNeasy kit (Qiagen).

QPCR (40–45 cycles per primer pair) was performed with Sybr Green Mastermix (Applied Biosystems) in an ABI 7500 machine. qPCR primers used were as follows:

Gfi1B: cttaccactgtgtcaagtgcaac, ctcctgtgagtggacgtgagtat.

Meis1: gccatacaagtgttaaggtttcatc, cctccttctctatcatctatcacaa.

Hoxa9: ccttatggcattaaacctgaacc, tgtttttctctatcaactggaggag,

Pbx1: ggacattttacagcaaattatgacc, cattaaacaaggcaggcttcattc,

Hoxb3: agttccacttcaaccgttatttgt, ccttctggtctttcttgtacttcat.

Hoxc4: atgatcatgagctcgtatttgatg, gactgtgttcagggatgtagctatt.

Hoxd13:agctcaaagaactggagaatgagta, cttggagacgattttcttgtcct.

P values of the qPCR data were calculated using one way Anova comparisons with Halm-Sidak post-hoc test using GraphPad Prism™ (Version 6) software.

### Plasmid Construction

Gfi-1b, and P2A-Gfi-1b expression vectors were as previously described [27], the Gfi1b-del5+6 vector was constructed by PCR amplification of Gfi1b sequences upstream of the 5^th^ zinc finger (corresponding to amino acids 1–272) followed by subcloning the product into the XbaI site of pEF1alpha. The Meis1 promoter constructs were produced by subcloning the indicated regions of the Meis1 promoter upstream of the luciferase gene in the pGL3 vector (Promega). The Gfi1b core promoter plasmid was a gift of T. Moroy.

### Cell Lines, Transfections and Luciferase Assays

MEL, a murine erythroleukemia cell line comprised of erythroid precursors arrested at the proerythroblast stage [37]; L8057, a megakaryoblastic cell line derived from a C3H/He mouse [38] and HEK-293T (ATCC # CRL-11268™) a transformed human kidney cell line were cultured as previously described [27]. MEL and L8057 cells were transduced with shRNA carrying retroviruses as indicated above.

Transfection experiments were performed with 50–70% confluent HEK-293T cells plated in wells of a 24 well plate which were co-transfected with 1 µg of the luciferase reporter and the indicated amounts of the expression vectors along with 50 ng of EF4-β-gal expression vector. Cells were harvested 48 hrs after transfection and lysed in 100 µl of CCLR lysis buffer (Promega). 20 µl of lysate was mixed with 100 µl of luciferase assay reagent (Promega) and luminescence measured on a Glorunner™ microplate luminometer (Turner Biosystems).

### Animal Welfare and Euthanasia

Animals (mice) were housed and bred in the CCNY vivarium (The City College/CUNY Medical School Institutional Animal Care and Use Committee number: A3733-01) as per the PIs approved animal protocol (#0858) and in accordance with USDA and institutional guidelines. For collection of embryos, pregnant female mice were euthanized by asphyxiation with carbon dioxide, delivered at less than 5psi per second. This study was approved by The City College IACUC.

### Harvesting and Culture of Fetal Liver Cells

Fetal liver cells were harvested from e12.5 embryos whose genotypes was confirmed as previously described [11]. Livers were dissociated by passaging through a 25G needle and syringe and ∼10^5^ cells were plated in IMDM medium with 20%FCS, supplemented with either 2 U/ml of erythropoietin and 20 ng/ml of SCF (stem cell factor) or with 20 ng/ml of thrombopoietin and 10 ng/ml of IL-3 and cultured for 5 days, followed by harvesting for total RNA.

## Results

### Lineage Specific *meis1* Regulation by LSD1

Chromatin immunoprecipitation screens (ChIP on chip) for common gene targets of Gfi1b, LSD1 and CoREST in erythroid cells revealed several (653) potential common transcriptional targets of all three factors [27]. To further investigate regulation of these targets in erythroid cells, expression profiling was performed in control versus LSD1 inhibited MEL (murine erythroleukemia) cells. The rationale for the screen being that the common targets of Gfi1b, LSD1 and Rcor1 should be transcriptionally derepressed in LSD1 deficient cells relative to controls, given that LSD1 functions as a transcriptional repressor in the context of Gfi1b and CoREST [27]. Of the 653 putative targets, *gfi1b* itself was most highly upregulated in LSD1 inhibited cells consistent with the established auto-regulation of this promoter [31]. The next most de-repressed message was found to be that of the homeo box protein, Meis1 (Table 1).

**Table 1:** Microarray profiling of Gfi1b and Meis1 expression in erythroid (MEL) cells upon LSD1 inhibition.

Gene	EX (lsd kd)	Fold increase	AccessionNo.	Full Name/Brief description
***gfi1b***	**3.340036542**	**10.1**	NM_008144	Growth factor independence 1b
***meis1***	**2.927793305**	**7.63**	NM_010789	Myeloid ecotropic viral integration site 1

Increase in mRNA levels of Gfi1b and Meis1 (fold increase) in LSD1 depleted MEL cells relative to controls.

**Figure 1:**
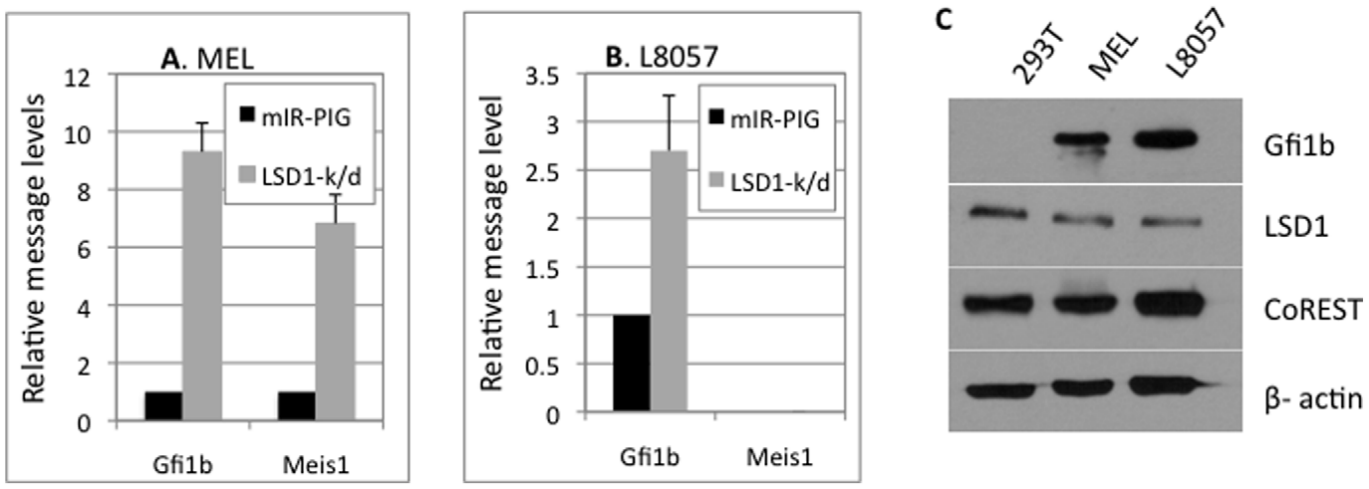
Message levels of Gfi1b and Meis1 in erythroid and megakaryocytic cells. Quantitative PCR (qPCR) of relative Gfi1b and Meis1 mRNA levels (normalized for HPRT) in (A) MEL (erythroid) and (B) L8057 (megakaryocytic) cells transduced with empty vector (mIR-PIG) or LSD1 shRNA (LSD1 k/d). Average of three experiments is shown, error bars represent standard deviation. C. Steady state protein levels of Gfi1b, LSD1 and CoREST relative to β-actin in HEK-293T, MEL and L8057 cells.

To assess repression of *meis1* by LSD1 in the context of Gfi1b, *meis1* expression and subsequent de-repression in cells deficient in LSD1, was determined by quantitative PCR (qPCR) analysis in the closely related erythroid and megakaryocytic lineages (Figure 1A and B). Both *gfi1b* and *meis1* were found to be upregulated in erythroid (MEL) cells deficient in LSD1 relative to controls. In contrast, *meis1* was neither expressed in control, nor upregulated upon LSD1 depletion, in the closely related megakaryocytic (platelet) lineage, L8057 cells. Given that both lineages express high levels of Gfi1b, LSD1 and CoREST (Figure 1c) and that *gfi1b* itself is upregulated upon LSD1 inhibition in both lineages, the *meis1* promoter appears to be specifically targeted for differential regulation by Gfi1b and its co-factors in these closely related lineages. Curiously, gene targeting experiments demonstrated an absolute requirement for *meis1* in megakaryopoiesis [39]. Therefore, the absence of Meis1 in this mouse megakaryoblastic line capable of differentiating into megakaryocytes *in vitro* [27], [38] along with its insensitivity to regulation by Gfi1b/LSD1/CoREST indicates stage specific inactivation of *meis1* in this lineage.

### Differential *meis1* Promoter Occupancy and Chromatin Status in Erythroid Versus Megakaryocytic Cells

**Figure 2:**
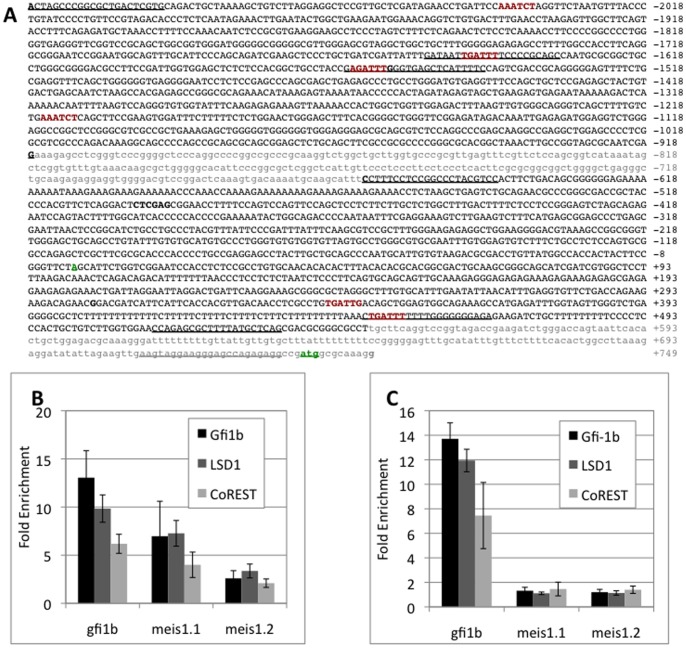
Occupancy of two distinct promoter regions of the *meis1* promoter by Gfi1b/LSD1/CoREST. **A.** 2.1 kb sequence of the murine Meis1 promoter and ∼0.75 kb of coding sequence spanning the two ∼1.2 kb segments (denoted in black uppercase lettering) obtained from ChIP on chip screening for Gfi1b/LSD1/CoREST targets. Intervening and downstream sequences not obtained from ChIP are indicated in grey lowercase letters. The putative transcriptional start site (indicated as +1 in the sequence) as inferred from the *meis1* transcript sequence (NM_010789) reported in the nucleotide database (also see text) and the initiator codon, (also according to the database) are indicated in green. The Gfi1b consensus elements are highlighted in bold maroon lettering. Sequences of primers used for ChIP qPCR or for amplification of promoter segments for subcloning are underlined (see also Materials and Methods). **B,C.** Chromatin immunoprecipitation (ChIP) analysis of the Gfi1b promoter and two Meis1 promoter segments (Meis1.1: distal; relative to tss and Meis1.2: promoter proximal) in erythroid and megakaryocytic lineages. Enrichment of the indicated promoter sequences relative to the immunoglobulin switch µ (Sµ) sequence in MEL (B) and L8057 (C) cells are indicated. Results shown are the average (solid bars) and standard deviations (error bars) of three independent experiments.

The original ChIP on chip experiments identified two distinct ∼1.2 kb long Meis1 promoter segments that hybridized to DNA sequences selectively enriched in Gfi1b/LSD1/CoREST immunopecipitates relative to immunoglobulin controls in MEL cells (Figure 2A). Both of these contained putative consensus and quasi-consensus Gfi1/1b binding sites. To define the locations of the Gfi1b, LSD1 and CoREST associated sites on the *meis1* promoter, ChIP experiments were conducted in MEL and L8057 cells. Although, the Meis1 promoter exhibits multiple distinct transcriptional start sites (tss), the majority of these transcripts originate at or near the point corresponding to the 5′ end of the mRNA designated NM_010789 in the NCBI data base. Therefore, for clarity we considered the 5′ end of NM_010789 as a representative tss and designated the ChIP sequences as distal (Meis1.1) and proximal (Meis1.2) relative to it. Although, we did not verify the actual tss for *meis1* in hematopoietic cells, we observed that both regions of the putative *meis1* promoter were occupied by Gfi1b, LSD1 and CoREST in MEL cells (Figure 2b). Of the two, the distal putative non-transcribed promoter region exhibited relatively greater affinity for Gfi1b/LSD1/CoREST compared to the proximal site, which overlapped with the transcribed region of the gene. However, at both sites, the pattern of enrichment of the promoter sequences in the immunoprecipitates exhibited a similar trend. Promoter sequence enrichment was highest for Gfi1b immunoprecipitates consistent with its direct binding to its specific DNA recognition element, intermediate for LSD1 and lowest for CoREST, reflecting the likely order in which these co-factors bind to DNA or chromatin via interaction with Gfi1b. Gfi1b recruits LSD1 to chromatin via its SNAG domain, which in turn associates with and brings CoREST to the locus [27].

In sharp contrast to erythroid cells, but consistent with the insensitivity of its promoter to LSD1 levels, neither region of the *meis1* promoter was occupied by any of these three factors in L8057 cells (Figure 2C) even though all three proteins were present in (Figure 1C), and associated with the *gfi1b* promoter (Figure 2C), in these cells. Therefore, the *meis1* promoter appears to be inaccessible to these factors at the megakaryoblast and subsequent stages of development of this lineage despite comparable levels of all three factors in both cell types (Figure 1C).

**Figure 3:**
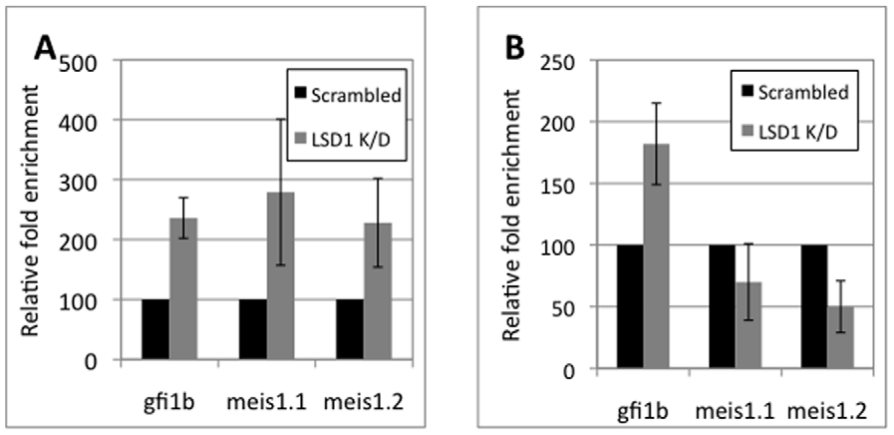
Di-methyl H3–K4 levels in *meis1* promoter chromatin. Chromatin immunoprecipitation (ChIP) analysis of the *gfi1b* promoter and two *meis1* promoter segments as indicated Figure 2A in erythroid and megakaryocytic lineages. Relative enrichment of the indicated promoter sequences for di-methyl H3–K4 in control (scrambled) versus LSD1 knocked down cells was calculated relative to that for immunoglobulin switch µ (Sµ) sequences in MEL (A) and L8057 (B) cells respectively. Results shown are the average (solid bars) and standard deviations (error bars) of three independent experiments.

In order to investigate the chromatin condition of the *meis1* promoter in the two lineages, particularly the methylation status of the H3–K4 residue, a substrate of LSD1 [27], in promoter associated chromatin, CHIP experiments were performed in both lineages. Both the *gfi1b* and *meis1* promoters showed comparable enrichment of di-meH3–K4 in MEL cells and further enhancement of this modification upon LSD1 inhibition (Figure 3A and data not shown). However in L8057 cells, the *gfi1b* promoter showed ∼10 fold greater di-meH3–K4 enrichment compared to the *meis1* promoter (not shown) which was further enhanced upon LSD1 depletion, while di-meH3–K4 levels at the *meis1* promoter remained very low and essentially unaltered upon LSD1 knock down (Figure 3B). These results confirm the silent and unresponsive state of the *meis1* promoter in L8057 cells, though the mechanism(s) responsible for it, and for precluding the recruitment of the Gfi1b protein complex to it in megakaryocytes, remain to be determined.

### Lineage Specific Deregulation of meis1 upon Loss of Gfi1b

**Figure 4:**
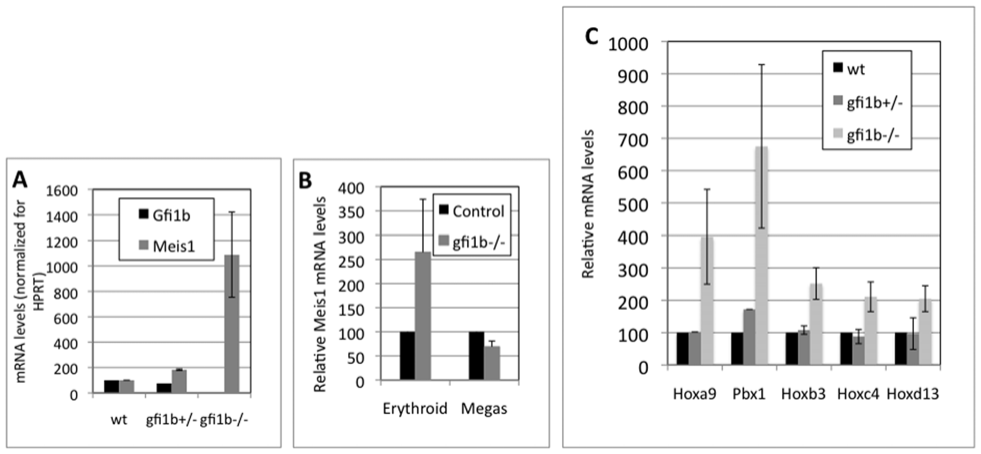
Message levels of Meis1, Pbx1 and Hox family members in fetal liver cells. **A.** QPCR of relative Gfi1b and Meis1 mRNA levels (normalized for HPRT) in embryonic day 12.5 (e12.5) wild type, *gfi1b+/−* and *gfi1b−/−* fetal liver cells. **B.** QPCR of relative Meis1 mRNA levels (normalized for HPRT) in control (wild type or *gfi1b+/−*) and *gfi1b−/−* fetal liver cells differentiated along the erythroid or megakaryocytic lineages *ex-vivo*. **C.** QPCR of relative Hoxa9, Pbx1, Hoxb3, Hoxc4 and Hoxd13 message levels from total fetal liver cells of the indicated genotypes. Averages and standard deviations from multiple embryos of different genotypes from three cohorts are shown. The p values for Pbx1, Hoxb3 and Hoxc4 levels in wild type versus mutant cells were <0.001 and those for Hoxa9 and Hoxd13 were <0.01. All values <0.05 were considered significant.

To confirm that *meis1* is a bona fide target of, and is regulated by, Gfi1b *in vivo,* its relative expression level was determined in wild type, heterozygous and *gfi1b* mutant fetal liver cells at day 12.5 of embryonic development (e12.5), since fetal livers consist mainly of erythroid progenitors in early and intermediate stages of differentiation at this age of embryogenesis [40]. Meis1 expression was found to be reciprocal to that of Gfi1b in e12.5 fetal liver cells, being significantly over-expressed in the mutants and slightly over-expressed in heterozygotes compared to wild type litter mates (Figure 4A).

Furthermore, even though haploinsufficiency of *gfi1b* consistently produced an ∼2 fold increase in Meis1 message in *gfi1b+/−* fetal livers (Figure 3A), this change in the level of Meis1 did not appear to produce any discernible phenotype in either fetal liver, or subsequent stages, of murine hematopoiesis [11]. To investigate lineage specific repression of the *meis1* promoter in primary cells, e12.5 fetal liver cells were differentiated along the erythroid or megakaryocytic lineages *ex vivo* by being cultured in erythropoietin (epo) and SCF or thrombopoietin (tpo) and IL-3 respectively, for 5 days (Figure S1). Interestingly, mutant cells cultured under erythroid conditions (with epo and SCF) continued to exhibit elevated *meis1* levels relative to controls (wild type or heterozygotes) although there was some diminution in the relative levels upon culture, probably due to the mutant cells adopting alternative cell fates (Figure 4B). In sharp contrast, progenitors from the same fetal livers, cultured under megakaryopoietic conditions (with tpo and IL-3) showed either no difference in relative *meis1* levels between control and mutant cells or even a slight decrease in the latter (Figure 4B). This proves that in primary cells, like in cell lines, *meis1* is selectively repressed only in erythroid cells by Gfi1b and not in megakaryocytes. Therefore, although *meis1* is expressed in primary megakaryocytic cells, its expression appears to be independent of Gfi1b.

### Deregulation of *Hox* Genes and *pbx1* in *gfi1b* Mutants

Meis1 is often co-ordinately over-expressed in various leukemias with its transcriptional partners the TALE domain protein Pbx1 and one or more homeodomain containing Hox family members, and collaborates with them in accelerating leukemogenesis [33], [41]. Moreover, Meis1, Hoxa9 and Pbx1 were also recently demonstrated to be upregulated in *gfi1* mutant bone marrow myeloid progenitors resulting in their hyper-proliferation [34].

These observations prompted an assessment of message levels of *pbx1* and certain *hox* genes in e12.5 day fetal livers from wild type, heterozygous and *gfi1b−/−* embryos. Specifically, the relative expression of messages encoding proteins known to either associate directly with Meis1 (Pbx1, Hoxa9 and Hoxb3) [33] or *hox* members known to be expressed in hematopoietic stem cells/progenitors during normal development (Hoxc4) [42] or ectopically expressed upon undergoing translocations in leukemias (Hoxd3) [43], from multiple *hox* clusters were interrogated. These messages exhibited substantial (Pbx1) to moderate (Hoxa9) to modest and consistent (Hoxb3, Hoxc4 and *Hoxd13*) upregulation in *gfi1b−/−* fetal livers relative to controls (Figure 4C). Since the *pbx* and *hox* gene promoters were not identified in the original ChIP screen, the mechanism responsible for the observed deregulation of these proteins in the absence of Gfi1b in fetal liver cells i.e either by loss of direct binding and de-repression of their promoters in *gfi1b−/−* cells or by other indirect means, remains to be determined.

### Repression of the Isolated *meis1* Promoter by Gfi1b

**Figure 5:**
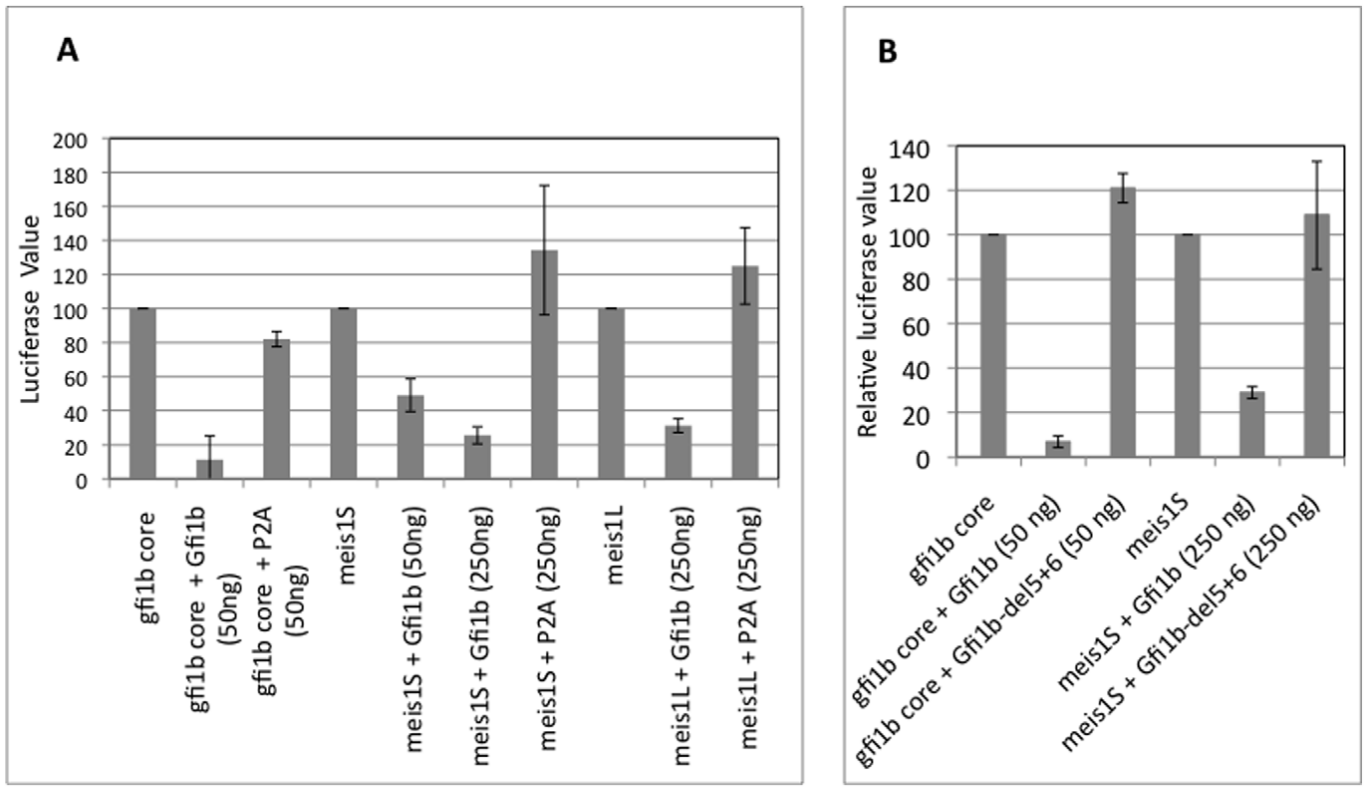
Regulation of the isolated *meis1* promoter by exogenous Gfi1b. Luciferase reporter based promoter assays in HEK-293T cells. 1 µg of reporter plasmid along with the indicated amount of expression plasmid and 50 ng of β-galactosidase expression vector was transfected into ∼10^6^ cells and assayed for luciferase activity. The 0.5 kb *gfi1b* core promoter was used as a positive control. The *meis1L* promoter consisted of 2.7 kb of promoter sequence from −2.1 kb to +0.65 kb (see Figure 2A) and the *meis1S* consisted of 1.25 kb of promoter sequence from −0.60 kb to +0.65 kb relative to the tss of *meis1*. P2A-represents the P2A-Gfi1b SNAG domain mutant; Gfi1b-del5+6-represents the Gfi1b deletion mutant lacking zinc fingers 5 and 6. For each promoter set, values shown are relative to that obtained in the absence of the corresponding expression vector, following normalization of all luciferase values for β-galactosidase levels. The average (solid bars) and standard deviations (error bars) from 3 independent experiments is shown.

To demonstrate direct Gfi1b specific repression of the isolated *meis1* promoter by Gfi1b, luciferase reporter based promoter assays were performed in the non-hematopoietic human embryonic kidney cell line HEK-293T. This line does not express any endogenous Gfi1 or Gfi1b but does express the relatively ubiquitous co-factors LSD1 and CoREST (Figure 1C). Two segments of the *meis1* promoter comprising 2.4 kb (Meis1L) and 1.2 kb (Meis1S) of sequences upstream of the initiator ATG codon (Figure 2A) were both repressed by Gfi1b in a dose dependent manner, but neither by the SNAG domain mutant P2A-Gfi1b nor (in case of meis1S) by the DNA binding deletion mutant Gfi1b-del5+6 which lacks the 5^th^ and 6^th^ zinc fingers (Figure 5), despite comparable expression of all recombinant proteins in these cells (Figure S2). These results shows that the isolated *meis1* promoter is repressed by Gfi1b in a SNAG domain and DNA binding dependent manner. Interestingly, despite the presence of multiple consensus Gfi1b binding elements, the Meis1 promoter appeared to be somewhat less responsive to Gfi1b protein levels relative to the 500 bp *gfi1b* core promoter itself (Figure 5) that had previously been shown to be auto- and cross- regulated by Gfi1b and Gfi1 respectively [31]. The *gfi1b* core promoter was maximally repressed by 50 ng of Gfi1b while the *meis1* promoter was only partially repressed by the same level of Gfi1b and needed five fold more Gfi1b to be completely repressed. This difference in the dose responsiveness of the two promoters toward Gfi1b protein levels may reflect their relative sensitivity *in vivo* to Gfi1b concentrations and is also consistent with the difference in their level of upregulation upon LSD1 inhibition in erythroid cells (Figure 1A). Although repression of the isolated *meis1* promoter by Gfi1 has not been similarly demonstrated in reporter assays, we predict that given the similarities between Gfi1 and Gfi1b wrt to sequence and binding site specificities [17], the results should be very similar. However, simply expression of one or both of these paralogs in any cell type, may not guarantee transcriptional regulation of *meis1* by them.

## Discussion

The results presented above demonstrate that the oncogene *meis1* is a bonafide target of Gfi1b and is repressed by this factor *in vitro* and *in vivo*. Since Gfi1b is required for both definitive erythropoiesis and megakaryopoiesis [11], and Meis1 for megakaryopoiesis in the fetal liver [39], repression of *meis1* by Gfi1b in fetal liver cells may appear paradoxical at first pass. However, a recent study demonstrating the ability of Meis1 to promote megakaryopoiesis at the expense of erythropoiesis in bone marrow MEPs [44], suggests the probability of a similar scenario in the fetal liver. Therefore, Gfi1b mediated repression of *meis1* in fetal liver MEPs likely ensures the channeling of the majority of these cells into the erythroid lineage, thereby ensuring the appropriate developmental stage specific equilibrium between these two lineages in this organ. Interestingly, our results demonstrate that the *meis1* promoter is refractile to Gfi1b and its cofactors in megakaryocytes despite high levels of the latter in this lineage. How the Gfi1b transcriptional complex is either specifically excluded from the *meis1* locus in megakaryocytes or conversely is selectively recruited to it in erythroid cells remains to be determined. In any case, our identification of *meis1* as a crucial transcriptional target of Gfi1b complements a previous study documenting regulation of *meis1* by its paralog Gfi1 [34], and establishes it as a common target of both Gfi proteins in hematopoiesis. Identification of *meis1* as a common gene target of both Gfi1 and Gfi1b coupled with their association with several common co-factors (LSD1, CoREST, HDACs etc.) [27] further augments the mechanistic basis for the observed physiological interchangeability between these factors during hematopoietic development [23].

### Increasing Complexity of Hox/Meis/Pbx and Gfi1/1b Interactions During Evolution

Recently, functional antagonism was uncovered between the fly ortholog of the Gfi proteins, Sens and those of Meis1 (Homothorax; Hth) and its Hox partners during fly neurogenesis [35]. This antagonism was further demonstrated to be conserved between Gfi1 and Meis1/Hoxa9/Pbx1 in mammalian hematopoiesis although the underlying mechanism was found to be different [34]. Our results now reiterate the conservation of this antagonism between the Meis/Hox/Pbx conglomerate and both Gfi paralogs during evolution from flies to mammals, despite the complex lineage diversification associated with mammalian hematopoiesis.

**Figure 6:**
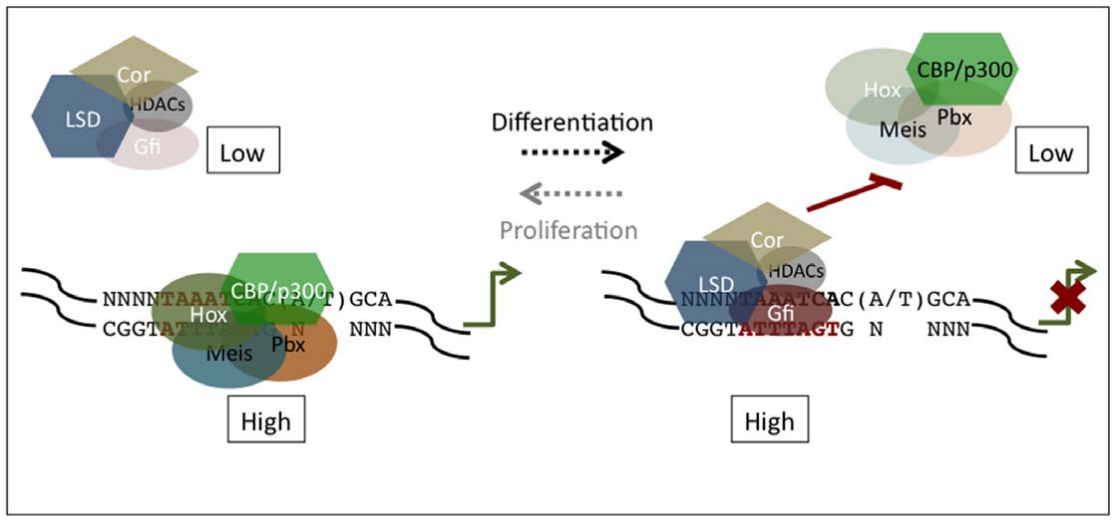
Bimodal mechanism of cell cycle inhibition by Gfi proteins. Transition from proliferation to commitment and/or differentiation (black arrow) follows an increase in Gfi1/1b concentration (“low” versus “high”, as indicated by increased shading). Increasing concentrations of Gfi1/1b repress *meis/hox/pbx* transcription and as levels of the latter decline (fading shapes) they may also be replaced on putative common promoters by Gf1/1b repressive complexes resulting in transcriptional inhibition of target genes. Ectopic expression of Meis/Hox/Pbx proteins in leukemias (grey arrow) may produce the opposite effect and reverse the normal differentiation program.

However, the molecular mechanism underlying *sens/Gfi* and *Meis/Hox/Pbx* antagonism has undergone a major modification from flies to mammals. In mammals, expression of the *hox* and *meis1* genes themselves have come under direct or indirect Gfi1/1b regulation, while in flies Sens and Hox/Meis complexes compete for binding to overlapping sequences in the promoters/enhancers of common target genes and accordingly repress or activate their transcription respectively, as demonstrated recently for the regulation of the *rhomboid* (*rho*) enhancer [35]. Whether this apparently newly acquired mode of *meis1/hox* repression by Gfi1/1b simply supplements and/or partially or entirely supplants physical competition between these proteins for binding to overlapping DNA recognition elements at common potential targets in the mammalian genome remains to be determined. Interestingly, the Sens and Gfi1/1b binding elements have been conserved between flies and mammals [45]. Moreover, analogous to the sequence overlap between the Sens and Hox/Hth binding sites in the *rhomboid* (*rho*) enhancer [35], the consensus Gfi1/1b and Hox/Meis1/Pbx1 binding sites that have been characterized in their respective mammalian targets also exhibit substantial sequence overlap [17], [30], [32]. Interestingly, the Hoxa9/Meis1/Pbx1 core consensus motif A**TGATTTA**TGGC [32] that was recently shown to be present on the majority of Hoxa9/Meis1 gene targets obtained by ChIP-Seq screening [46] is a perfect reverse complement of the core Gfi1/1b consensus motif **TAAATCA**C(A/T)GCA [17], [30]. Thus, similar competition between Gfi1/1b and Hox/Meis1/Pbx1 for binding to gene regulatory elements in the mammalian genome is a likely possibility if these binding sites or loci are accessible to, or otherwise able to recruit, these opposing sets of factors. Therefore, we hypothesize that Gfi1/1b may antagonize Hox/Meis/Pbx function in mammals in a bimodal manner by simultaneously or sequentially binding to and reducing the transcription of Hox, Meis1 and Pbx1 factors while also stoichiometrically displacing them from other downstream targets common to both sets of proteins (Figure 6). Since Hox/Meis/Pbx proteins recruit CBP^p300^, a histone acetyl transferase, to their target sites thereby activating their transcription [46], the displacement of these activating complexes with the repressive Gfi/LSD1/CoREST/HDAC complex(es) on the promoters of cell cycle promoting genes likely initiates the transition from proliferation to commitment and/or differentiation of progenitors poised for maturation along different lineages. Conversely, in leukemias, ectopic over-expression of wild type or fusion Meis/Hox/Pbx proteins may displace Gfi1/1b repressive complexes from the promoters of such genes leading to their over-expression (Figure 5). Although several known Hox/Meis/Pbx targets such as *Pim1*, c-*myb*, *c-myc*, *CD34*, *flt3* and *cycD* [34], [41] could likely mediate this effect, the verification of the above model requires either demonstration that the above genes are also common targets of Gfi1/1b and/or the identification and characterization of other similar common transcriptional targets. If this bimodal mechanism of regulation is, in fact, observed in mammals and not in flies then it would represent an additional level of fine tuning gene expression by Gfi proteins that was acquired during mammalian evolution.

### Dual Roles of Gfi1/1b in Hematopoiesis

Over-expression of Gfi1 and Gfi1b have been observed in induced and naturally occurring lymphoid and myeloid leukemias in mice and humans respectively [9], [13], [14], [15], [16], [17], [18], [19], [21]. However, the causal role of these genes if any in initiation or maintenance of these leukemias is not clear. In contrast, the results presented here in combination with recent evidence [34], [47] indicate that Meis1 and its oncogenic partners Pbx1 and Hoxa9 are actively repressed by both Gfi1 and Gfi1b in multiple lineages and demonstrate that these proteins indeed exhibit growth inhibitory, tumor suppressor-like properties. Accordingly, deletion of either *gfi1* or *gfi1b* results in hyper-proliferation of hematopoietic stem and progenitor cells [1], [2], [3], [4], [11]. Moreover, recently a hypomorphic mutant of *gfi1* was found to be responsible for predisposing individuals bearing this mutation to AML by deregulating Hoxa9 expression in their myeloid cells [47]. This same mutant was also found to collaborate with K-RAS in inducing fatal myeloproliferative disease in mice [47]. Therefore, these erstwhile “oncogenes” seem to function more like tumor suppressors under normal circumstances, and their oncogenic propensities if any, may be exposed upon corruption of their normal function, perhaps under the influence of other oncogenes, during leukemogenesis.

In conclusion, this study demonstrating the repression of the *meis1* promoter in a lineage specific manner by Gfi1b establishes this oncogene as a crucial common target of mammalian Gfi proteins. These results together with that demonstrating antagonism between their orthologs in flies [35], highlights an evolutionarily ancient mechanism for regulating gene expression that has been essentially conserved from flies to mammals while acquiring increased sophistication and complexity in the latter, in accordance with their expanded lineage repertoires.

## Supporting Information

Figure S1 **Cell morphology of wild type and** ***gfi1b−/−*** **fetal liver cells cultured** ***ex vivo*** **.** Phase contrast images of e12.5 fetal liver cells from wild type and mutant embryos as indicated, cultured with epo and SCF (top panel) or tpo and IL-3 (bottom panel). Erythroid differentiation is indicated by the presence of small differentiated erythrocytes in the top left image, while megakaryocytic differentiation is evidenced from the presence of large megakaryocytes in the bottom left image. *gfi1b−/−* mutants do not give rise to either lineage [11].
(TIF)

Figure S2 **Expression of recombinant proteins in 293T cells.** Western blot showing expression of the indicated recombinant proteins following transfection of their expression vectors into 293T cells. ∼60 µg of total protein from whole cell lysates was loaded in each lane.
(TIF)
