# Supplementary figures and images for: Differential Transcriptional Regulation of meis1 by Gfi1b and Its Co-Factors LSD1 and CoREST
Source: PLoS One. 2013 Jan 7;8(1):e53666. doi: 10.1371/journal.pone.0053666 (PMC3538684; doi:10.1371/journal.pone.0053666)

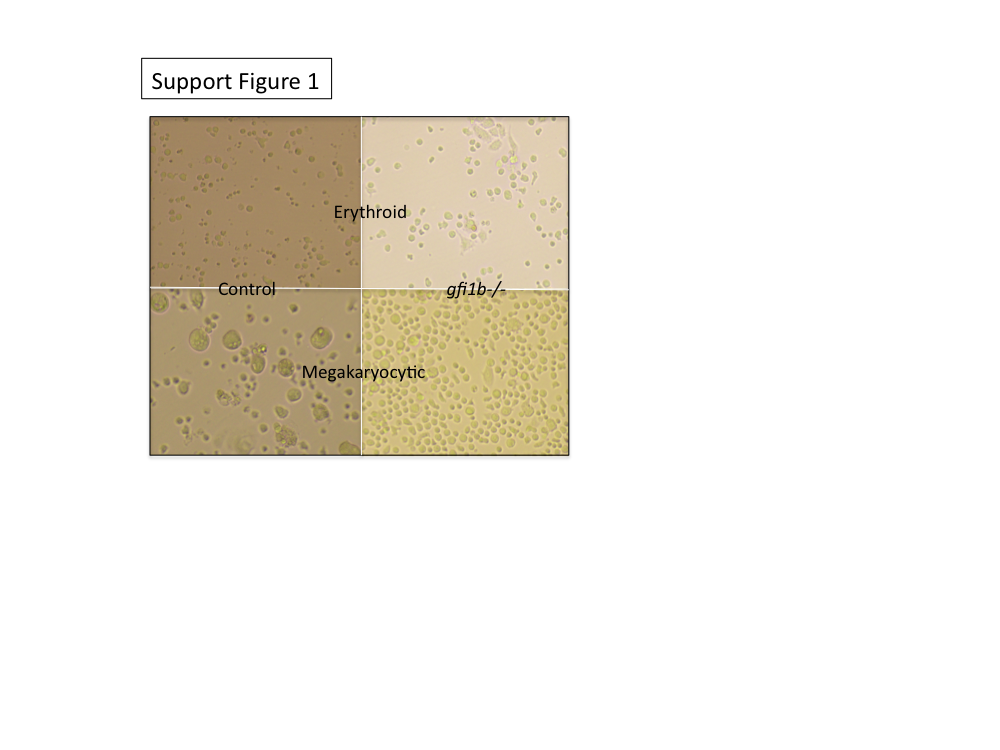

Supplement: Figure S1 — Cell morphology of wild type and gfi1b−/− fetal liver cells cultured ex vivo . Phase contrast images of e12.5 fetal liver cells from wild type and mutant embryos as indicated, cultured with epo and SCF (top panel) or tpo and IL-3 (bottom panel). Erythroid differentiation is indicated by the presence of small differentiated erythrocytes in the top left image, while megakaryocytic differentiation is evidenced from the presence of large megakaryocytes in the bottom left image. gfi1b−/− mutants do not give rise to either lineage [11]. (TIF) [file pone.0053666.s001.tif]
